# Optimal tuning of PI based LF for three-phase SRF PLL synchronization system using pity beetle algorithm under grid abnormalities

**DOI:** 10.1038/s41598-025-03530-6

**Published:** 2025-05-29

**Authors:** P. Riyas, S. A. Lakshmanan

**Affiliations:** Department of Electronics and Communication Engineering, Amrita School of Engineering, Amrita Vishwa Vidyapeetham, Chennai, India

**Keywords:** Engineering, Electrical and electronic engineering

## Abstract

The importance of grid synchronization in recent years is primarily driven by the widespread integration of renewable energy sources (RES). As nations transition toward carbon–neutral power systems, the variable nature of renewables and the increasing prevalence of non-linear loads introduce significant challenges for maintaining effective grid synchronization. At the core of this process, the synchronous reference frame phase lock loop (SRF PLL) has become a significant component of grid connected power electronic converters (PEC). Generally, PLL includes a proportional integral (PI) based loop filter (LF) that plays a critical role in ensuring precise phase angle and frequency alignment between the grid and PEC. Traditional PI controller tuning techniques like the symmetrical optimum (SO), optimum setting algorithm (OSA) and Ziegler-Nichol’s exhibit satisfactory performance under ideal grid conditions. However, their effectiveness diminishes in real-world scenarios characterized by grid disturbances. To overcome these limitations, this paper proposes a novel PI tuning approach based on the pity beetle algorithm (PBA) for the SRF PLL grid synchronization system. Inspired by the foraging character of the pityogenes chalcographus beetle, PBA optimizes the PI parameters of the LF, enhancing accurate synchronization and response speed. The proposed method is thoroughly assessed under challenging grid abnormalities such as harmonic distortion, amplitude fluctuations, phase jump and unbalanced phase differences. The SRF PLL with proposed PBA tuning for PI based LF is mathematically formulated and analyzed through numerical simulations using MATLAB tool. A comprehensive stability analysis is conducted through frequency response bode plots to validate the effectiveness of the tuning method for accurate grid synchronization. The results, encompassing phase margin (PM), accuracy, computational cost and adaptability derived from the suggested PBA tuned LF design for SRF PLL are compared with existing tuning methods.

## Introduction

**Fig. 1:**
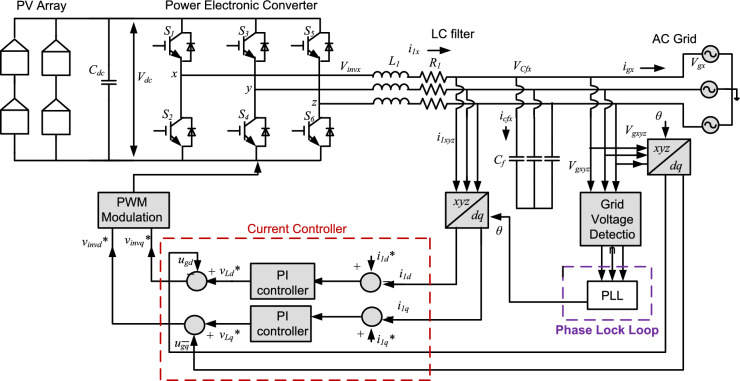
Grid connected PV system with current controller and synchronization unit.

Advancement in the field of renewable energy sources (RES) and distributed generation (DG) based power systems have led to an increased deployment of Power Electronics Converters (PEC) to function as an active interface circuit between the AC power grid, ensuring efficient and reliable operation^1^. Integration of RES demands a periodic revision of Grid Code Requirements (GCR) provided by international bodies and national authorities^2^. This is due to the continuous enhancement and diversification of grid side converters (GSC) with additional capabilities and features to support the grid and improve power quality. Generally, GSC shows complex and fast transient behaviour in the presence of fault conditions or disturbances. Therefore, to provide precise responses and adherence to modern grid standards, the GSC control algorithms must operate effectively under both stable and disturbed grid conditions such as harmonics, phase jump, voltage imbalance, frequency deviation and swell/sags^3^. The basic control structure of a Photovoltaic (PV) interfaced three-phase AC power grid system is shown in Fig. 1. Control of a GSC primarily comprises two components: the synchronization unit and the internal current control loop^4^. Real/reactive power controller determines reference currents that the inner current controller trails to inject the necessary active and reactive power. The synchronization unit extracts phase angle and frequency, which are subsequently utilized inside the control loops. Thus, a reliable grid synchronization technique is essential for accurately detecting the fundamental frequency component of the grid voltage and phase angle while efficiently filtering out harmonics under non-ideal grid conditions and generating precise reference currents^5^.

**Fig. 2:**
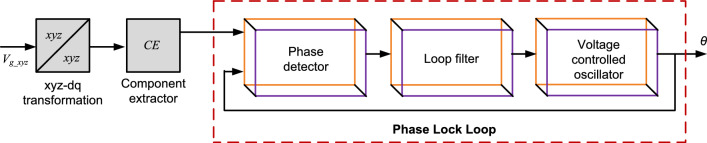
Functional block diagram of PLL.

Accurate and stable synchronization of grid-tied systems, such as inverters with the utility grid ensures smooth power transfer and system stability. Among the various techniques used for grid synchronization, the Phase Locked Loop (PLL) has emerged as a widely adopted method due to its robustness and simplicity^6^. Basically, PLL consists of key components including the phase detector (PD), loop filter (LF), and voltage controlled oscillator (VCO)^7^. Figure 2 demonstrates the functional block diagram of the PLL and the primary function of the PLL is to effectively ascertain and sustain the frequency, phase relationship, and amplitude synchronization between the system and grid. Numerous efforts have been made to enhance the controllers within the PLL to effectively track grid parameters. Synchronous reference frame (SRF) based PLL is considered as one the best PLL designs for grid synchronization under ideal grid voltage states. However, when the grid conditions evolved greatly over the last few years and attempts were made to improve the tuning of the and Proportional Integral (PI) based LF of existing SRF PLL to make it adaptable for non-ideal grid conditions^8^.

Traditional methods for PI tuning in PLL systems, such as the Ziegler-Nichols, Symmetrical Optimum (SO), Cohen-Coon, Optimum Setting Algorithm (OSA) and trial-and-error approaches, have historically served as the foundation for controller design^9,10^. These methods rely on heuristic or empirical rules to determine the PI controller gains based on the system response to step or frequency disturbances^11^. While relatively simple to implement, these techniques often assume a linear and time-invariant system, which limits their applicability in complex, dynamic environments like modern power grids^12^. The Ziegler-Nichols method involves setting gains based on the system’s critical oscillation point which can result in suboptimal performance under high harmonic distortion or abrupt phase jumps^13^. Similarly, the Cohen-Coon method, though effective for certain process control applications, may struggle to provide adequate robustness or speed of response in nonlinear grid synchronization scenarios^14^. Fuzzy logic based PI tuning is an intelligent method that dynamically adjusts its controller parameters based on the disturbances in the grid voltage^15^. However, computational complexity, stability and dependence on rule-based design limit its capability in PI tuning for complex grid environments. Among the noteworthy contributions in this domain, the JAYA algorithm has been applied to optimize PI tuning^16^ in SRF PLL and Unified Power Quality Conditioners (UPQC). Additionally, a Particle Swarm Optimization (PSO) based PI tuning approach has been proposed to enhance hydrogen fuel efficiency in fuel cells^17,18^.

A neural network based tuning strategy referred to as a Neural Tuning Machine (NTM) has been developed for PI controllers used in regulating active and reactive power in wind energy conversion system^19^. The Snake optimizer algorithm has been employed in a PD-PI cascaded controller for accurate speed control of brushless DC motors^20,21^. Similarly, a Golden Eagle optimizer has been utilized to design a PI controller of fractional order, particularly for power factor correction converters^22^. Furthermore, an OSA has been introduced and this technique demonstrates superior performance compared to traditional SRF PLL tuning methods^23^. Another notable development is PI tuning using the Whale Optimization Algorithm (WOA) that can improve the Total Harmonic Distortion (THD) and other time domain characteristics. After a thorough analysis of the existing literature, the following research gaps have been identified.

- Conventional tuning methods increase the bandwidth, leading to disturbances and multiple harmonic elements in the estimated parameters.
- Traditional PI controller tuning approaches rely on fixed parameter settings, which are inadequate for grids with a high penetration of variable RES.
- Conventional tuning methods depend on complex mathematical models, and the extracted frequency and phase angle generate harmonic ripples, leading to an increase in the THD of the grid voltage signal.

In recent years, nature inspired optimization algorithms have gained significant attention for their ability to handle nonlinear and multidimensional challenges^24^. This paper proposes the implementation of Pity Beetle Algorithm (PBA) for tuning the PI parameters of an LF present in the SRF PLL to improve grid synchronization performance. The PBA, a swarm intelligence algorithm modelled after the hunting behaviour of the Pityogenes chalcographus beetle, offers an effective balance between exploration and making it well-suited for complex optimization problems^25^. By applying the PBA to the PLL tuning process, the objective is to achieve improved dynamic response and robustness under various grid disturbances. With the optimized PI parameters, the proposed method is mathematically modelled, evaluated through frequency response analysis, and tested in harmonic distortion, amplitude fluctuations, phase jump and unbalanced phase differences in the grid voltage signals. The results demonstrate the effectiveness of PBA in enhancing synchronization accuracy, reducing settling time, and minimizing phase errors, even under challenging grid conditions. This study provides a novel approach to tune the PI controller for grid synchronization, leveraging bio-inspired algorithms to meet the demands of modern power systems. This research offers the following major contributions.

- The proposed PBA tuning model demonstrates superior performance compared to existing PI tuning methods, making it highly suitable for integrating variable large-scale RES. It offers moderate computational complexity, fast response times, and exceptionally low settling times, ensuring efficient operation.
- The model is tested under various non-ideal grid scenarios, including phase jumps, harmonic distortions, unbalanced grid voltages, and phase imbalances, consistently achieving exceptional synchronization of the source with the grid.
- The developed model precisely tracks frequency and phase angle accurately under diverse grid conditions

This paper is structured as follows: Mathematical model of the SRF PLL system for grid synchronization is discussed in “Mathematical modelling of synchronous reference frame PLL (SRF PLL)” section In “Pity beetle algorithm (PBA)” section, the proposed PBA tuning for PI controller based LF for SRF PLL and the process involved to extract the controller gains for accurate estimation frequency, phase angle is discussed. Stability analysis to validate the effectiveness of the proposed PI controller tuning is explored in “Stability analysis of SRF PLL using conventional and proposed tuning methods” section. The obtained results and comparative analysis of the actual and estimated phase, phase angle, and frequency for both existing and proposed PI tuning methods under four different grid abnormalities are presented in “Results and discussion” section. Finally, “Conclusions” ends with a conclusion of this research paper.

## Mathematical modelling of synchronous reference frame PLL (SRF PLL)

**Fig. 3:**
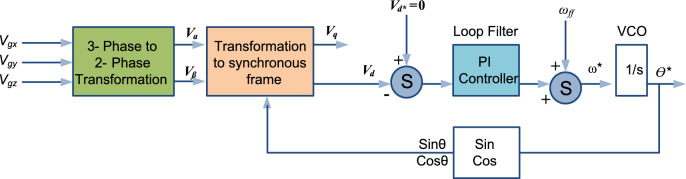
Mathematical control structure of three-phase SRF PLL system.

SRF PLL is one of the widely accepted PLL for three-phase systems and its mathematical control structure is shown in Fig. 3. Basically, SRF PLL leverages a rotating reference frame aligned with the grid’s frequency, enabling it to accurately track the changes in phase and frequency. This capability benefits modern grids, where integrating RES introduces significant fluctuations. Input three-phase voltages are transformed into the $dq$ reference frame using Clark’s and Park’s transformations. These transformations convert the three-phase signals into two orthogonal components, simplifying the system analysis and control^26^.

A feedback loop within the SRF PLL continuously adjusts the angular position of the $dq$ frame, ensuring that the $q$-axis component is maintained at zero. This process is necessary for achieving precise synchronization between the system and the grid, allowing for stable operation even under non-ideal conditions. The three-phase grid voltages are represented as:

1 $${V}_{gx}={V}_{m}sin\theta$$

2 $${V}_{gy}={V}_{m}\text{sin}\left(\theta -\frac{2\pi }{3}\right)$$

3 $${V}_{gz}={V}_{m}\text{sin}\left(\theta +\frac{2\pi }{3}\right)$$

Here, ${V}_{gx}$, ${V}_{gy}$, and ${V}_{gz}$ represents the three-phase grid voltages, while $\theta$ denotes the phase angle. To facilitate the analysis, these voltages undergo Clark’s transformation to convert them into the $\alpha \beta$ frame, followed by Park’s transformation to yield the $dq$ frame. The transformation equation is defined as follows:

4 $$\left[\begin{array}{c}{V}_{\alpha }\\ {V}_{\beta }\end{array}\right]=\frac{2}{3}\left[\begin{array}{ccc}1& \frac{-1}{2}& \frac{-1}{2}\\ 0& \frac{-\sqrt{3}}{2}& \frac{\sqrt{3}}{2}\end{array}\right]\left[\begin{array}{c}{V}_{gx}\\ {V}_{gy}\\ {V}_{gz}\end{array}\right]$$

The transformation to the $dq$ frame is achieved using the following matrix

5 $${T}_{qd}=\left[\begin{array}{cc}{sin\theta }^{*}& {cos\theta }^{*}\\ {-cos\theta }^{*}& {sin\theta }^{*}\end{array}\right]$$

Applying this transformation and the resulting voltages in the $dq$ frame is given by:

6 $${V}_{q}={V}_{m}\text{cos}\left(\theta -{\theta }^{*}\right)$$

7 $${V}_{d}={-V}_{m}\text{sin}\left(\theta -{\theta }^{*}\right)$$

where ${\theta }^{*}$ is the calculated phase angle of the PLL system. The PI regulator is tuned such that, it tracks the phase angle $\theta$ accurately and $q$-axis component is driven to zero^26^. When the space vector is synchronized, the estimated frequency locks with the reference frequency of the system and the tracked phase angle ${\theta }^{*}$ and $\theta$ also matches. The open-loop transfer function model of the SRF PLL is formulated as below.

8 $${G}_{openloop}\left(s\right)={V}_{m}*\frac{1}{s}*\left({k}_{p}\frac{1+s{\tau }_{i}}{s{\tau }_{i}}\right)\left(\frac{1}{1+s{T}_{s}}\right)$$

Once the PI parameters are designed the closed-loop transfer function is represented as:

9 $$\begin{array}{*{20}c} {G_{closedloop} \left( s \right) = \frac{{G_{openloop} \left( s \right)}}{{1 + G_{openloop} \left( s \right)}}} \\ \end{array}$$

**Fig. 4:**
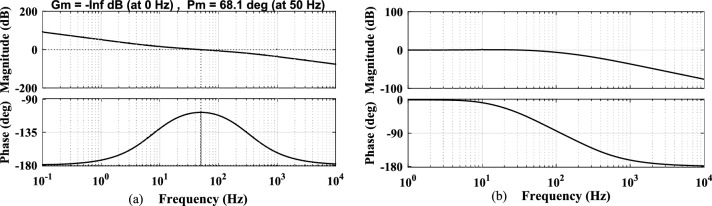
Bode plot analysis of Conventional SO method-based PI tuning for SRF PLL (**a**) open-loop response (**b**) closed loop response.

The PI controller based LF present in the SRF PLL is tuned by using the conventional SO technique and its open-loop/closed loop frequency response Bode plots are shown in Fig. 4.

The obtained Phase Margin (PM) is 68.1 degrees, which falls within the acceptable range. However, the high bandwidth increases oscillations in the extracted phase angle, frequency and develops specific harmonic contents under various non-ideal grid voltage conditions^23^. Moreover, the limitations of the conventional PI tuning method are evident that it affects the system’s stability and results in a slow dynamic response while tracking the phase angle and frequency. Given that grid synchronization demands the highest level of accuracy and speed, it is motivated to design an appropriate optimal PI tuning method for the SRF PLL system to achieve proper synchronization under grid abnormalities. The proposed PBA based PI tuning is discussed in the following sections to effectively accommodate the variability of RES and ensure reliable operation under non-ideal grid conditions. Furthermore, the PBA based PI tuning method aims to enhance the robustness of the SRF PLL system by dynamically adjusting the controller parameters to mitigate the adverse effects of grid disturbances.

## Pity beetle algorithm (PBA)

The Pity Beetle Algorithm (PBA) is an optimization technique put forward by Nikos et al. based on the scavenging and reproductive behaviour of the bark beetle, Pityogenes chalcographus^27^. This algorithm is modelled after the beetle’s life cycle, which includes three key phases: search, aggregation, and anti-aggregation. In the search phase, beetles seek out suitable trees for inhabiting and reproducing^28^. Upon finding a suitable tree, the beetles release pheromones to attract others during the aggregation phase, forming a colony. Once the colony reaches a certain size, the beetles release anti-aggregation pheromones to limit further growth, ensuring enough resources are available for the colony’s survival. For the numerical implementation of PBA, a randomly initiated population is distributed across the search space. The particles representing beetles then move through this space, searching for optimal positions. As they do so, new generations of solutions are formed, replacing older ones as their positions and velocities are updated. This process continues until the algorithm meets the termination criteria. PBA excels in solving complex optimization problems by efficiently exploring multidimensional search spaces to determine optimal solutions^29^.

A key strength of PBA is its ability to balance exploitation and exploration. Exploration allows the beetles to investigate new areas of the solution space, preventing them from getting trapped in local optima. Exploitation, meanwhile, focuses on refining promising solutions already discovered, pushing the search toward global optima. This balance is especially important when dealing with complex landscapes that contain multiple local optima, where traditional algorithms often struggle. The PBA also adapts its search behaviour based on proximity to optimal solutions, mimicking how real beetles adjust their movement when approaching a food source. Additionally, in some variations, multiple beetles collaborate in a swarm-like fashion, similar to Particle Swarm Optimization (PSO), to accelerate convergence by sharing information about potential solutions^30^.

### Modelling of the proposed PBA based PI tuning for SRF PLL grid synchronization

**Fig. 5:**
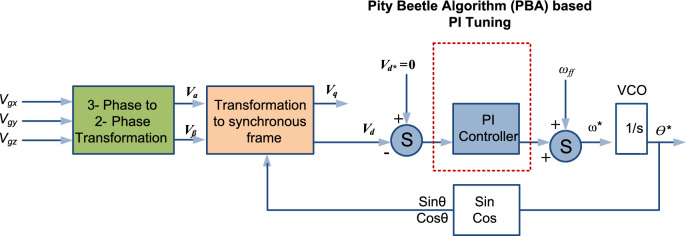
Mathematical control framework model of the proposed PBA based PI tuning for SRF PLL system.

The mathematical control framework model of the proposed PBA based PI tuning for SRF PLL grid synchronization is illustrated in Fig. 5. The main objective of the suggested method is to refine the PI parameters of LF in the SRF PLL and the transfer function is given as:

10 $$\begin{array}{*{20}c} {G_{PI} \left( s \right) = k_{p} \left( {1 + \frac{1}{{\tau_{i} s}}} \right)} \\ \end{array}$$

where ${k}_{p}$ is the proportional gain and ${\tau }_{i}$ is the integral time constant. In the proposed design, plant is modelled as a second order system and it is denoted as:

11 $$\begin{array}{*{20}c} {G_{plant} \left( s \right) = \frac{{V_{m} }}{{\left( {1 + \tau_{1} s} \right)\left( {1 + \tau_{2} s} \right)}}} \\ \end{array}$$

where ${V}_{m}$ is the amplitude of the input voltage, ${\tau }_{1}$ and ${\tau }_{2}$ are time constant approximated as ${\tau }_{1}={\tau }_{2}=\frac{{T}_{s}}{2}$; where ${T}_{s}$ denotes the sampling time. As the PLL system inherently experiences delay due to computation and hardware limitations, a delay term is added to the transfer function by Padé approximation. This approximation accurately models the time delay caused during signal processing, resulting in a better representation of the dynamic behaviour of the system. With these inherent delays taken into consideration, the system’s performance and stability can be analysed and optimized. The ${G}_{delay}$ is expressed as:

12 $$\begin{array}{*{20}c} {G_{delay} \left( s \right) = \frac{{\left( {1 - \frac{{T_{s} }}{2}s} \right)}}{{\left( {1 + \frac{{T_{s} }}{2}s} \right)}}} \\ \end{array}$$

The open loop transfer function is derived by multiplying the above equations and given as:

13 $$\begin{array}{*{20}c} {G_{openloop} \left( {\text{s}} \right) = {\text{G}}_{{{\text{PI}}}} \left( {\text{s}} \right)*{\text{G}}_{{{\text{plant}}}} \left( {\text{s}} \right)*{\text{G}}_{{{\text{delay}}}} \left( s \right)} \\ \end{array}$$

14 $$\begin{array}{*{20}c} {G_{openloop} \left( {\text{s}} \right) = k_{p} \left( {1 + \frac{1}{{\tau_{i} s}}} \right)*\frac{{V_{m} }}{{\left( {1 + \tau_{1} s} \right)\left( {1 + \tau_{2} s} \right)}}*\frac{{\left( {1 - \frac{{T_{s} }}{2}s} \right)}}{{\left( {1 + \frac{{T_{s} }}{2}s} \right)}}} \\ \end{array}$$

From the open loop equation, the closed loop transfer function can be derived as:

15 $$\begin{array}{*{20}c} {G_{closedloop} \left( s \right) = \frac{{G_{openloop} \left( s \right)}}{{1 + G_{openloop} \left( s \right)}}} \\ \end{array}$$

To optimize the PI parameters, a fitness function is defined as follows:

16 $$\begin{array}{*{20}c} {J\left( {k_{p} ,\tau_{i} } \right) = \left( {w_{1} *\theta_{error} } \right) + \left( {w_{2} *T_{settling time} } \right) + ({\text{w}}_{3} *\sum max(0,Re\left( {{\text{poles}}_{{{\text{closedloop}}}} } \right)} \\ \end{array}$$

where ${\theta }_{error}$ is the phase deviation $and {T}_{settling time}$ is the settling time, $\text{Re}({\text{poles}}_{\text{closedloop}})$ checks the stability of the closed loop transfer function by analysing the poles. ${w}_{1}$,${w}_{2}$ and ${w}_{3}$ are the weights assigned to each criterion, and their values are considered as 0.4, 0.3 and 0.3, respectively. The above fitness function is optimized using PBA algorithm to iteratively update the values of ${k}_{p}$ and ${\tau }_{i}$ as given below.

17 $$\begin{array}{*{20}c} {k_{p}^{{\left( {t + 1} \right)}} = k_{p}^{t} + d_{kp} \delta_{kp} } \\ \end{array}$$

18 $$\begin{array}{*{20}c} {\tau_{ip}^{{\left( {t + 1} \right)}} = \tau_{i}^{t} + d_{{\tau_{i} }} \delta_{{\tau_{i} }} } \\ \end{array}$$

where ${d}_{kp}$ and ${d}_{{\tau }_{i}}$ are the step sizes that control how much ${k}_{p}$ and ${\tau }_{i}$ change per iteration and ${\delta }_{kp}$ and ${\delta }_{{\tau }_{i}}$ are the random perturbations used for introducing randomness. To speed up the computation and avoid instability, limits are enforced for ${k}_{p}$ and ${\tau }_{i}$ as given below.

19 $$\begin{array}{*{20}c} {0.1 < k_{p} < 10} \\ \end{array}$$

20 $$\begin{array}{*{20}c} {0.001 < \tau < 1} \\ \end{array}$$

The initial sensing lengths ${d}_{kp}$ and ${d}_{{\tau }_{i}}$ are set to 0.1 and 0.01 whereas and step sizes ${\delta }_{kp}$ and ${\delta }_{{\tau }_{i}}$ are set to 0.01 and 0.001 respectively. The step-by-step process followed in the proposed PBA optimization algorithm for tuning the PI controller gains for SRF PLL grid synchronization is shown in Algorithm 1.

**Algorithm 1.**
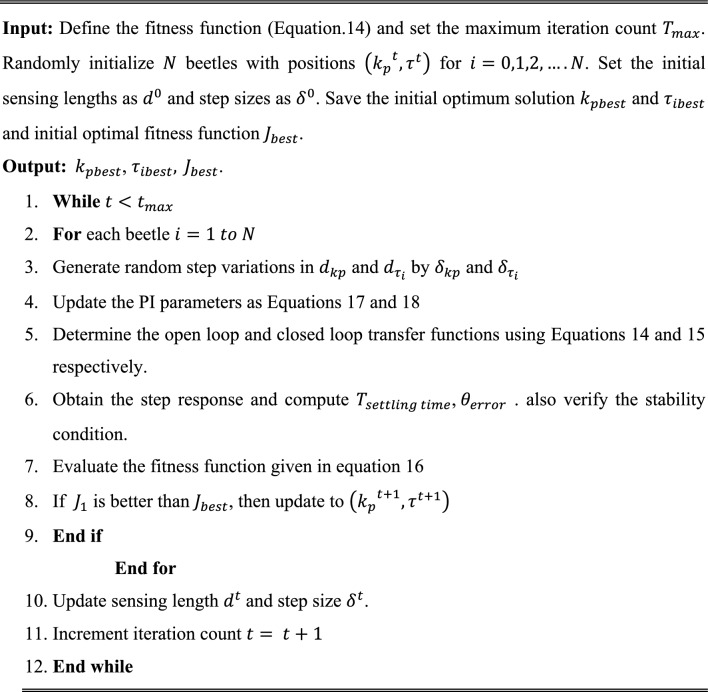
PBA based PI controller tuning for SRF PLL grid synchronization system

The system parameters utilized to efficiently address the tuning of controller gains using the proposed PBA method for SRF PLL grid synchronization are shown in Table 1. The algorithm uses population size of 30 and runs for a maximum of 50 iterations ${(T}_{max}$ = 50). The PBA optimized values are ${k}_{p}=$ 0.4554 ${\tau }_{i}=$ 0.0359. The PI controller gains from the proposed method and conventional method systematically analyzed and summarized in Table 2.

**Table 1:** System specifications.

System parameters	Values
Grid voltage	415 v
Frequency	50 Hz
Sampling time	0.5 ms

**Table 2:** PI controller gains derived by various tuning methods.

PI controller tuning methods for SRF PLL system	PI controller gains	
	${k}_{p}$	${\tau }_{i}$
Conventional SO technique	0.7545	0.0126
Conventional OSA method	0.4838	0.00803
Proposed PBA based PI tuning method	0.4554	0.0359

## Stability analysis of SRF PLL using conventional and proposed tuning methods

**Fig. 6:**
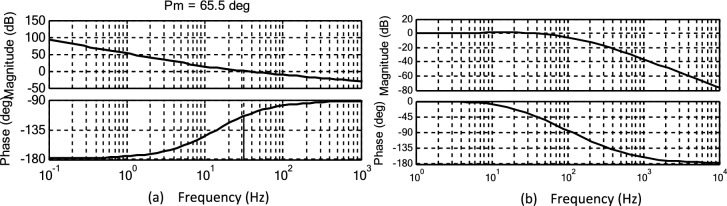
Frequency response analysis of conventional OSA based PI tuning for SRF PLL (**a**) open-loop response (**b**) closed loop response.

**Fig. 7:**
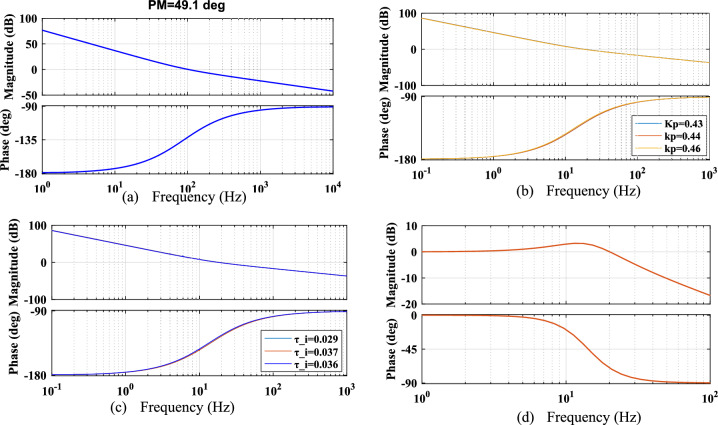
Frequency response analysis of proposed PBA based PI tuning for SRF PLL (**a**) open-loop response (**b**) response of variations in controller gain ${k}_{p}$ (**c**) response of variations in controller gain ${\tau }_{i}$ (**d**) closed loop response.

Stability enhancement is a critical aspect of system analysis when determining the controller gains using a proposed method, ensuring the robustness and reliability of the tuning approach under various non-ideal conditions. This section describes the stability assessment of the SRF PLL system under harmonic distortion, amplitude fluctuations, phase jump and unbalanced phase differences. Quantitative analysis of frequency response characteristics using Bode plots to examine the performance of the conventional, proposed PBA based PI tuning for the SRF PLL system are discussed. First, the Bode plot of the open-loop and closed loop transfer function of SRF PLL using the controller are gains derived from the conventional OSA is shown in Fig. 6. It is noted that, PM of the system is 65.5 degrees. However, increased bandwidth introduced harmonics in the tracked output signals in presence of grid abnormalities. The Bode response of the open-loop transfer function of the SRF PLL grid synchronization system using PBA tuning is shown in Fig. 7a. The response shows that system PM is reached at 49.1 degrees and all such increased order harmonic components are completely rejected as compared with traditional tuning approaches for PI based LF in the SRF PLL technique. Moreover, the proposed tuning method extends the required Bandwidth under non-ideal grid voltage signals and allows only fundamental frequency component.

**Fig. 8:**
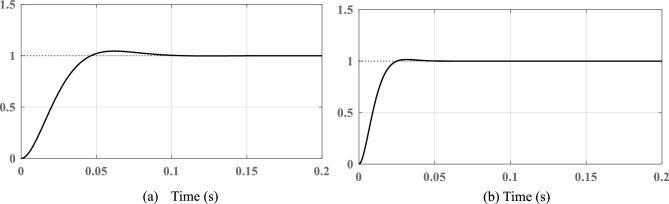
Closed loop step-response analysis (**a**) conventional tuning method (**b**) proposed PBA based PI controller tuning.

Mostly, stability enhancement depends upon controller gains and the working ability of the PBA based tuning is also tested under the variations in the derived controller gains. The frequency response of the system transfer function is simulated with the change of controller gains using the PBA tuning approach and the results are depicted in Fig. 7b,c respectively. It is observed that, in both cases, the system guarantees stability and produces the required PM/Bandwidth. The developed PBA tuning method is also tested under the closed-loop condition and the corresponding Bode plot is shown in Fig. 7d. As seen, PLL can work very close to the minimum frequency range and depicts low-pass filter characteristics. The proposed tuning method for SRF PLL effectively attenuates the peak at 0 dB in the low-frequency region. Extracted PM values from the traditional and developed tuning methods are summarized in Table 3. Step response analysis of an SRF PLL system is carried out using the controller gains derived from the PBA algorithm, and results are shown in Fig. 8. Time-domain specifications including, settling time, Peak overshoot and steady-state error are fairly reduced compared with conventional approaches. Moreover, the performance is improved significantly due to the optimal tuning of control parameters. The proposed method ensures enhanced dynamic response by minimizing transient deviations and reducing oscillations. The reduction in overshoot and settling time contributes to faster synchronization under grid disturbances.

**Table 3:** Extracted phase margin (PM) values from various tuning methods.

PI controller tuning methods for SRF PLL system	PI controller parameters		Extracted phase margin (PM) (in degrees)
	${k}_{p}$	${\tau }_{i}$	
Conventional SO technique	0.7545	0.0126	68.1
Conventional OSA method	0.4838	0.00803	65.5
Proposed PBA based method	0.4554	0.0359	49.1

## Results and discussion

Performance results of the PBA PI tuning method implemented in this research work for SRF PLL grid synchronization are analysed under four different non-ideal grid voltage cases as discussed below. The obtained results are evaluated against the classical SO tuning approach, Genetic algorithm (GA)^31^, Particle Swarm Optimisation (PSO)^17^ and Grey Wolf Optimisation (GWO)^32^ to examine the accurate tracking of phase angle and frequency. For metaheuristic algorithms, the same objective function is minimized. However, changes are made in population size, number of iterations, weights, and few other parameters to evaluate their impacts.

### Case 1: Presence of harmonics in the grid voltage

**Fig. 9:**
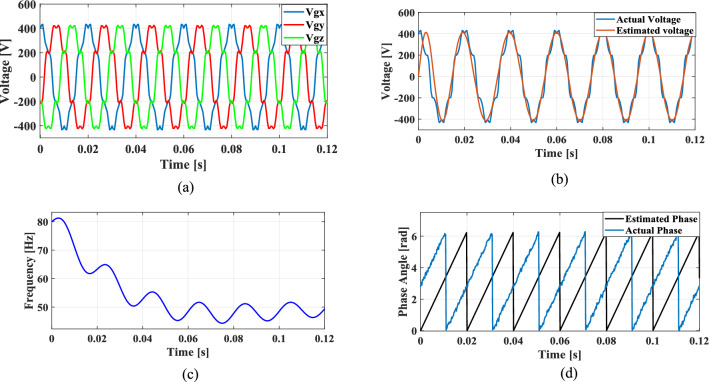
SRF PLL with conventional SO tuning (**a**) grid voltage signals with harmonics (**b**) estimated voltage (**c**) extracted frequency (**d**) extracted phase angle.

**Fig. 10:**
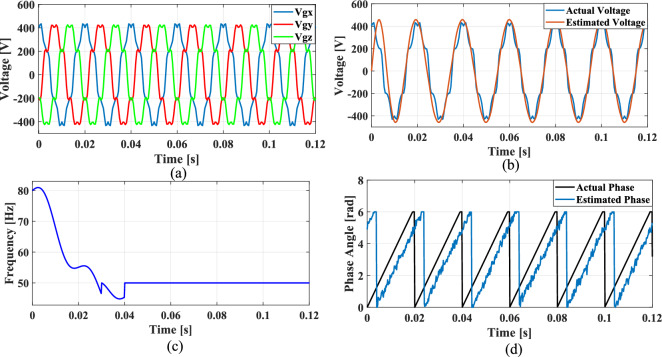
SRF PLL with GA tuning (**a**) grid voltage in the presence of harmonics (**b**) estimated voltage (**c**) extracted frequency (**d**) extracted phase angle.

**Fig. 11:**
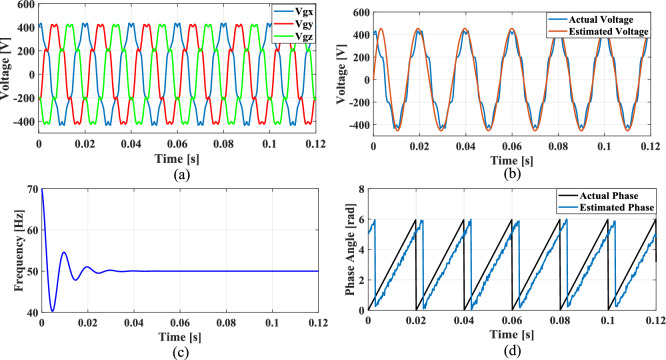
SRF PLL with PSO tuning (**a**) grid voltage in the presence of harmonics (**b**) estimated voltage (**c**) extracted frequency (**d**) extracted phase angle.

**Fig. 12:**
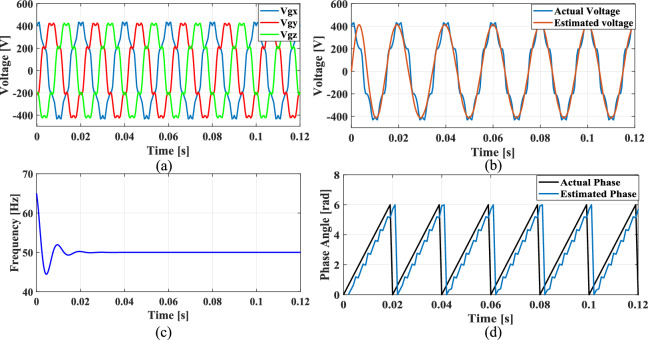
SRF PLL with GWO tuning (**a**) grid voltage with harmonics (**b**) estimated voltage (**c**) extracted frequency (**d**) extracted phase angle.

**Fig. 13:**
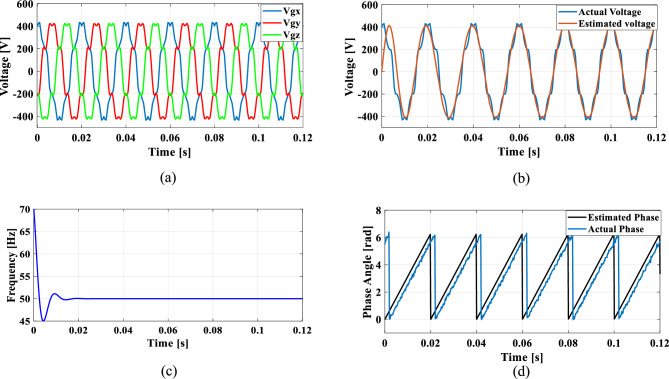
SRF PLL with proposed PBA based PI tuning (**a**) grid voltage signals with harmonics (**b**) estimated voltage (**c**) extracted frequency (**d**) extracted phase angle.

The non-linear loads introduce harmonics into the grid and to simulate this condition, 5th, 7th, and 11th harmonics were incorporated into the grid voltage at levels of 10%, 7%, and 5%, respectively. Figure 9a shows the grid voltage input with harmonics and Fig. 9b–d presents the corresponding extracted voltage, frequency and phase angle using the conventional SO tuning approach. Moreover, Figs. 10, 11, 12 depicts the results of estimated voltage, extracted frequency and phase angle using GA, PSO, GWO based PI tuning methods. Figure 13a–d demonstrates the respective performance results of the proposed PBA based PI tuned SRF PLL system. It is observed that the SO methods struggle to track frequency while the GA, PSO and GWO methods track frequency with initial oscillations.

The GWO method shows minimized settling time and slightly improved phase tracking accuracy among conventional methods followed by PSO. In contrast, the proposed PBA method is ahead of all the conventional methods with a settling time of 0.02 s and the highest level of accuracy in phase tracking. Additionally, the actual and estimated voltage aligns well and demonstrates the superior performance of the PBA PI tuning. These results emphasize the enhanced performance of the proposed PBA PI tuning method executed in this research work.

### Case 2: Unbalanced grid voltage

**Fig. 14:**
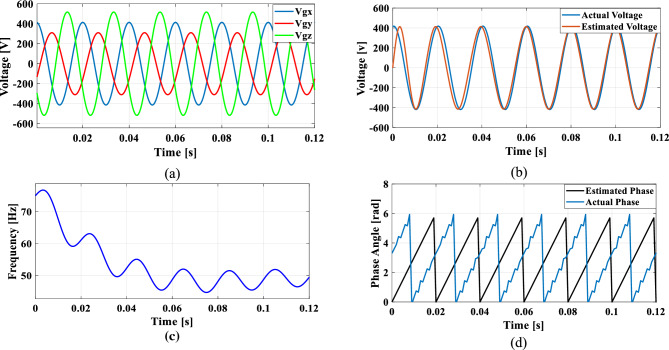
SRF PLL with conventional SO tuning (**a**) unbalanced grid voltage (**b**) estimated voltage (**c**) extracted frequency (**d**) extracted phase angle.

**Fig. 15:**
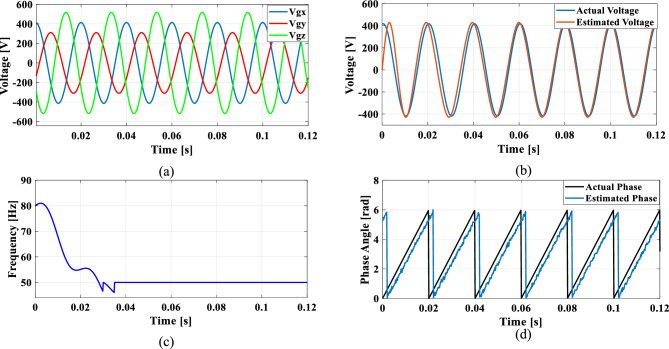
SRF PLL with GA tuning (**a**) unbalanced grid voltage (**b**) estimated voltage (**c**) extracted frequency (**d**) extracted phase angle.

**Fig. 16:**
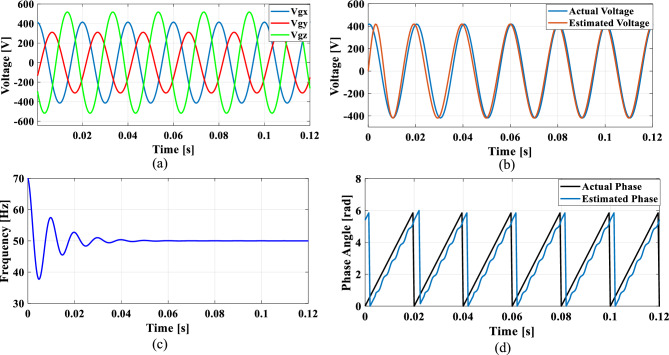
SRF PLL with PSO tuning (**a**) unbalanced grid voltage (**b**) estimated voltage (**c**) extracted frequency (**d**) extracted phase angle.

**Fig. 17:**
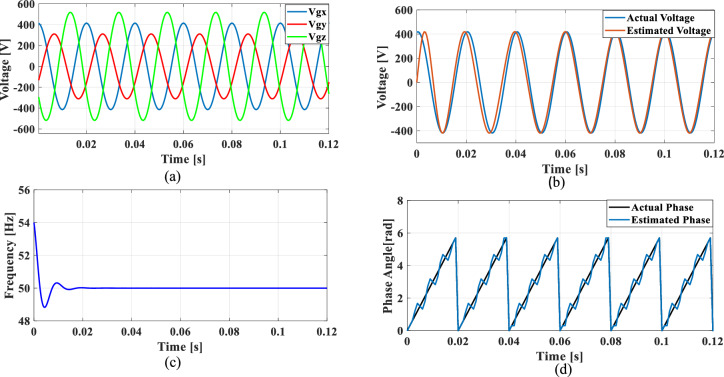
SRF PLL with GWO tuning (**a**) unbalanced grid voltage (**b**) estimated voltage (**c**) extracted frequency (**d**) extracted phase angle.

**Fig. 18:**
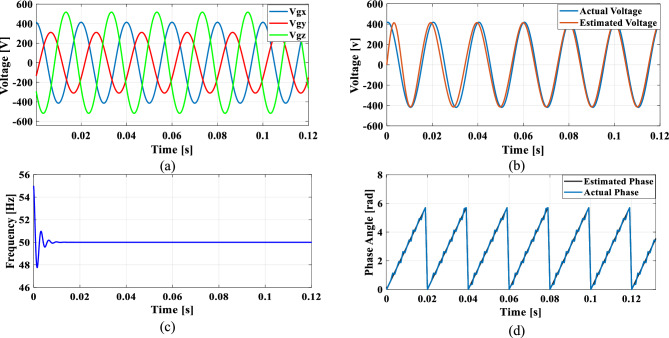
SRF PLL with proposed PBA tuning (**a**) unbalanced grid voltage (**b**) estimated voltage (**c**) extracted frequency (**d**) extracted phase angle.

Unbalanced load is a common occurrence in the grid and to explore this case, an unbalanced voltage scenario is created in which the voltage of Phase A is nominal, Phase B voltage is reduced by 25% than Phase A, and Phase C voltage is increased by 25% than Phase A. Figure 14 illustrates the SRF PLL under unbalanced grid voltage along with the corresponding tracked voltage, frequency, and phase angle using the existing SO tuning method. Similarly, Figs. 15, 16 and 17 shows the results of corresponding tracked voltage, frequency, and phase angle using GA, PSO and GWO techniques. Performance of the proposed PBA based PI tuned SRF PLL system are presented in Fig. 18a–d respectively.

From the obtained results, it is seen that, the proposed model achieves a substantially lower overshoot in frequency tracking with a remarkable fast settling time of 0.014 s. The GWO method has lower overshoot than proposed method however, with a higher settling time of 0.022 s. The proposed keeps balance between overshoot and settling time which would be an optimal method for PI tuning. The GA and PSO methods have suboptimal tuning comparing to the proposed method. Moreover, the conventional SO tuned model struggles with frequency estimation, as the PI regulator fails to settle at the final frequency value.

### Case 3: Phase jump in grid voltage

Phase jumps can occur due to grid faults, switching operations, line outages or connection or disconnection of large loads or generators. To analyze the functionality of the PLL under sudden load changes, a phase jump is introduced at 0.085 s, resulting in a sudden 10% voltage dip.

**Fig. 19:**
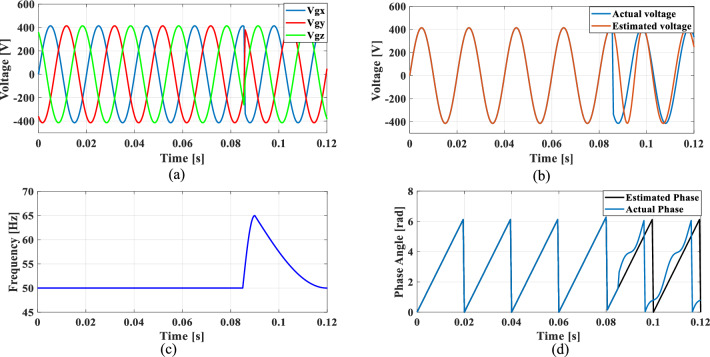
SRF PLL with conventional SO tuning (**a**) phase jump in the grid voltage (**b**) estimated voltage (**c**) extracted frequency (**d**) extracted phase angle.

**Fig. 20:**
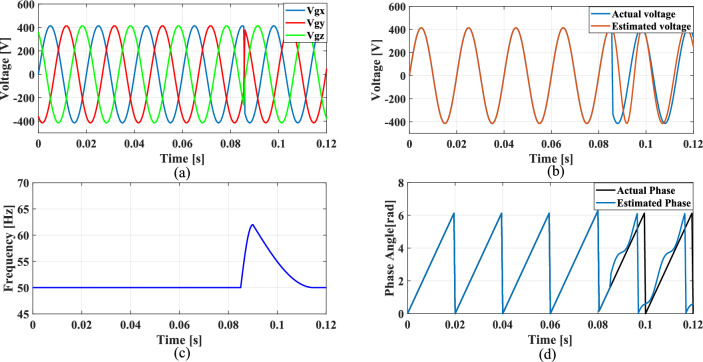
SRF PLL with GA tuning (**a**) phase jump in the grid voltage (**b**) estimated voltage (**c**) extracted frequency (**d**) extracted phase angle.

**Fig. 21:**
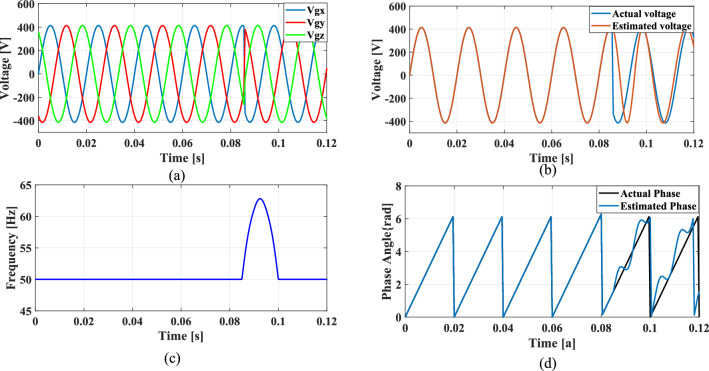
SRF PLL with PSO tuning (**a**) phase jump in the grid voltage (**b**) estimated voltage (**c**) extracted frequency (**d**) extracted phase angle.

**Fig. 22:**
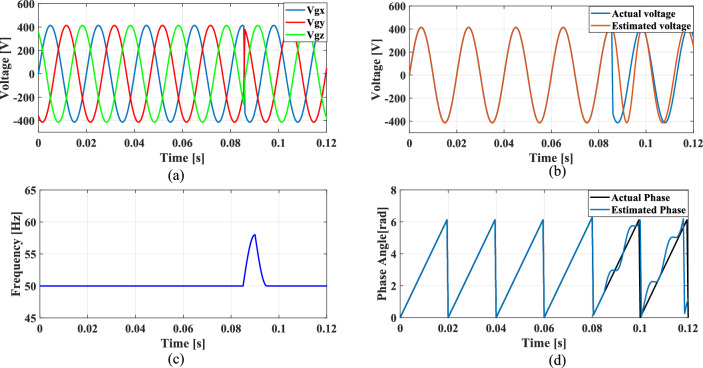
SRF PLL with GWO tuning (**a**) phase jump in the grid voltage (**b**) estimated voltage (**c**) extracted frequency (**d**) extracted phase angle.

**Fig. 23:**
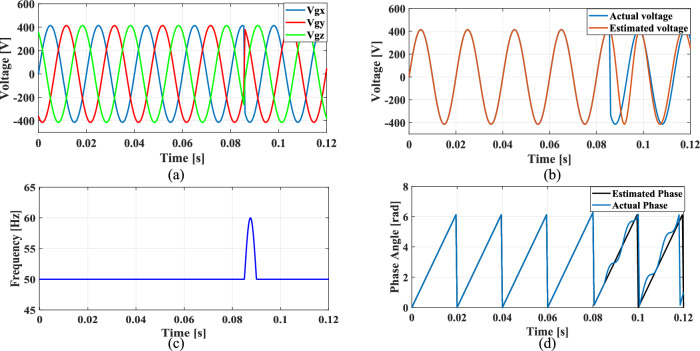
SRF PLL with proposed PBA tuning (**a**) phase jump in the grid voltage (**b**) estimated voltage (**c**) extracted frequency (**d**) extracted phase angle.

Figure 19a shows the grid voltage input with the phase jump at 0.085 s and Fig. 19b–d illustrates the corresponding estimated voltage, frequency and phase angle using the conventional SO tuning. Figures 20, 21, 22 and 23 depict the corresponding results of conventional GA, PSO, GWO and proposed PBA based PI tuning methods. The effectiveness of the PBA tuning method in addressing phase jumps is noteworthy and the model accurately tracks the frequency by detecting a phase jump at 0.085 s, with correction implemented by the PI regulator at 0.088 s.

The conventional methods take a long time to settle with less accuracy in phase tracking. GWO shows better accuracy in detecting the phase jump followed by PSO. The PI controller implemented the correction at 0.088 s for the proposed PBA method followed by GWO at 0.095 s and PSO at 0.1 s respectively. Overall, the proposed method demonstrates greater stability, accurate phase angle tracking and exhibits improved dynamic response compared to the conventional tuning approach under the phase jump in the grid voltage signal.

### Case 4: Unbalanced phase difference

In a balanced three-phase system, the phase differences between each phase are typically maintained at 120 degrees. However, unbalanced loading and various grid conditions can lead to deviations in these phase angles. To simulate such scenarios, where the phase difference between phase A and phase B is reduced by 10%, while the phase difference between phase A and phase C is increased by 10%.

**Fig. 24:**
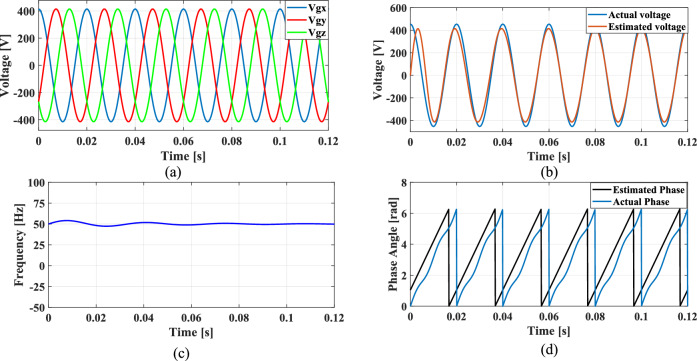
SRF PLL with SO tuning (**a**) unbalanced phase difference in the grid voltage (**b**) estimated voltage (**c**) tracked frequency (**d**) tracked phase angle.

**Fig. 25:**
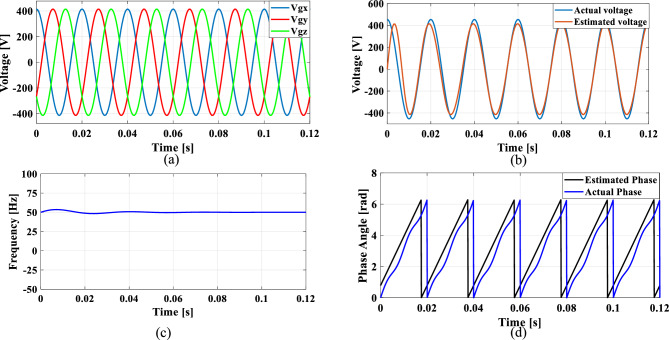
SRF PLL with GA tuning (**a**) unbalanced phase difference in the grid voltage (**b**) estimated voltage (**c**) tracked frequency (**d**) tracked phase angle.

**Fig. 26:**
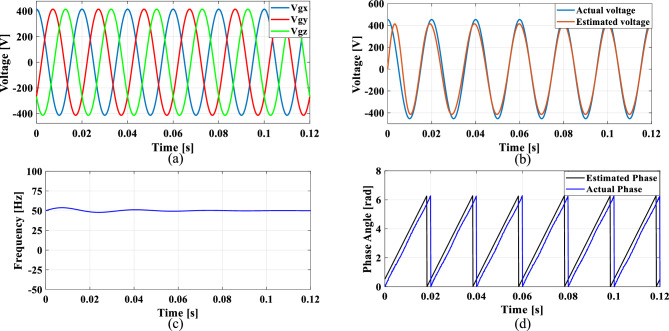
SRF PLL with PSO tuning (**a**) unbalanced phase difference in the grid voltage (**b**) estimated voltage (**c**) tracked frequency (**d**) tracked phase angle.

**Fig. 27:**
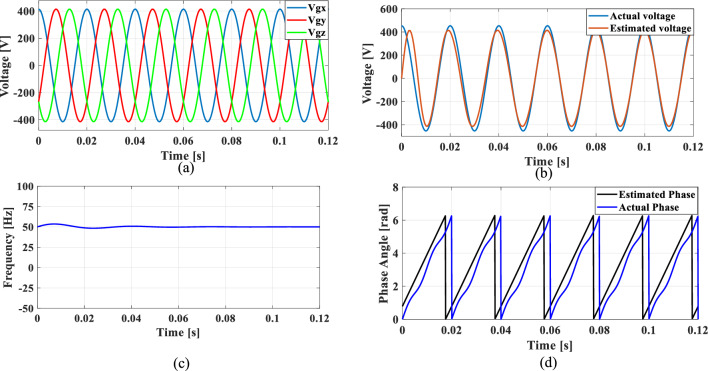
SRF PLL with GWO tuning (**a**) unbalanced phase difference in the grid voltage (**b**) estimated voltage (**c**) tracked frequency (**d**) tracked phase angle.

**Fig. 28:**
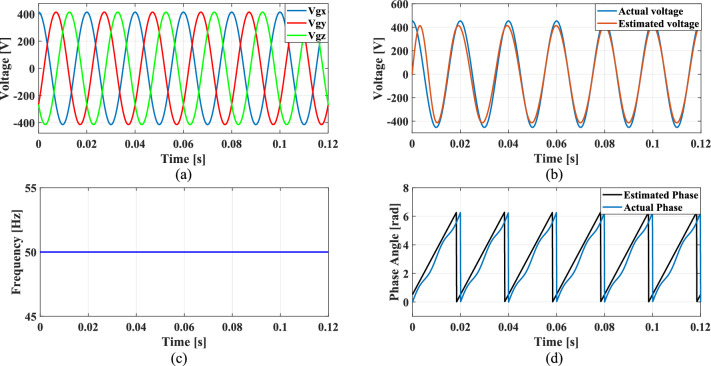
SRF PLL with proposed PBA tuning (**a**) unbalanced phase difference in the grid voltage (**b**) estimated voltage (**c**) tracked frequency (**d**) tracked phase angle.

Figures 24, 25, 26 and 27 illustrate the SRF PLL under unbalanced phase difference in the grid voltage along with the estimated voltage, frequency, and phase angle for conventional SO tuning, GA, PSO and GWO tuning methods respectively. Additionally, Fig. 28 shows the respective results for the proposed PBA based tuning approach. The proposed PBA based PI tuning method for grid synchronization demonstrates enhanced accuracy in estimating both phase angle and frequency. Unlike other test cases, it tracks frequency very accurately with no time delay. This highlights the efficiency of the proposed method in enhancing performance metrics in phase estimation.

Achieving a phase margin of 49.1 degrees, the method exhibits stability across a wide range of both optimal and suboptimal grid conditions. Furthermore, the settling time of the newly introduced method is significantly better than that of existing PLL methods, highlighting its improved performance in dynamic environments. A Comprehensive performance analysis of conventical PI tuning methods including SO, GA, PSO, GWO and the proposed PBA based PI tuning for SRF PLL Grid synchronization system are summarized in Table 4.

**Table 4:** Comprehensive performance analysis of conventical PI tuning methods including SO, GA, PSO, GWO and the proposed PBA based PI tuning for SRF PLL Grid synchronization systems.

Grid abnormalities	Performance metrics	SO based tuning method	GA tuning method	PSO tuning method	GWO tuning method	Proposed PBA tuning method
Harmonics in the grid voltage: 5th, 7th and 11th harmonics at levels of 10%, 7%, and 5%,	Extracted Frequency settling time (sec)	0.12	0.04	0.036	0.026	0.02
	Accuracy in estimated phase angle	Less accurate	Less accurate	Medium accurate	accurate	More accurate
	Reduced transient enhancement	Low	Low	Moderately fast	Moderately Fast	Fast
Unbalanced grid voltage: voltage of Phase A is nominal, Phase B voltage is reduced by 25%, and Phase C voltage is increased by 25%	Extracted Frequency settling time (sec)	0.12	0.036	0.05	0.022	0.014
	Accuracy in estimated phase angle	Less accurate	Less accurate	Medium accuracy	Medium accurate	More accurate
	Reduced transient enhancement	Low	Low	Moderately fast	Fast	Fast
Phase jump at 0.85 s in the input grid signal	Extracted Frequency settling time (sec)	0.12	0.118	0.1	0.095	0.088
	Accuracy in estimated phase angle	Less accurate	Less accurate	Medium accuracy	Medium accurate	More accurate
	Reduced transient enhancement	Low	Low	Low	Fast	Fast
Unbalanced phase difference	Oscillations in extracted frequency	High	High	Low	Low	Low
	Accuracy in estimated phase angle	Less accurate	Medium accuracy	Accurate	Accurate	More accurate
	Reduced transient enhancement	Low	Low	Fast	Fast	Fast

## Conclusions

Accurate grid synchronization under normal, abnormal grid voltage conditions is an essential part for reference current generation and executing various closed loop control algorithms. Basically, SRF PLL is the most adapted method for grid synchronization applications and tuning of PI based LF decides the exact values phase angle, frequency. This paper presents an optimal PI tuning approach based on the Pity Beetle Algorithm (PBA) for enhancing the performance of the SRF PLL grid synchronization system for grid connected power electronic converters applications. Unlike conventional PI tuning techniques such as SO, OSA, and Ziegler-Nichols, which exhibit limited adaptability under real world grid disturbances, the proposed PBA based tuning strategy demonstrates superior robustness in handling dynamic grid abnormalities. The mathematical formulation and numerical simulations validate the effectiveness of the proposed method, with key performance improvements observed in terms of phase margin, synchronization accuracy, and transient response are discussed. The frequency response analysis using Bode plots is carried out to confirm the enhanced stability of the proposed tuning approach, particularly in mitigating the effects of harmonic distortions, amplitude variations, and phase imbalances. Moreover, comparative results highlight the computational efficiency of PBA in achieving optimal PI parameters for SRF PLL grid synchronization system. The obtained results exhibit the potential of bio-inspired optimization techniques for real-time grid synchronization applications, providing a viable alternative to conventional tuning strategies.

## Data Availability

The datasets used and/or analysed during the current study available from the corresponding author on reasonable request.
